# Effectiveness of Knowledge Translation Strategies for Guideline Implementation: Protocol for an Update of an Overview of Systematic Reviews

**DOI:** 10.1002/gin2.70058

**Published:** 2026-01-28

**Authors:** Kian Torabiardakani, Shraddha Sawant, Gonzalo Bravo‐Soto, Daniela Montalva‐Romero, Rachel J. Couban, Abigail E. Chu, Norman Buckley, Gordon Guyatt, David Juurlink, Jason W. Busse, Andrea J. Darzi

**Affiliations:** ^1^ Department of Anesthesia McMaster University Hamilton Ontario Canada; ^2^ Michael G. DeGroote National Pain Center McMaster University Hamilton Ontario Canada; ^3^ Department of Health Research Methods, Evidence, and Impact McMaster University Hamilton Ontario Canada; ^4^ Michael G. DeGroote Institute for Pain Research and Care McMaster University Hamilton Ontario Canada; ^5^ Faculty of Health University of Waterloo Waterloo Ontario Canada; ^6^ Department of Medicine University of Toronto Toronto Ontario Canada

**Keywords:** clinical practice guidelines, implementation strategies, knowledge translation, systematic reviews

## Abstract

**Background:**

Healthcare guidelines are necessary to optimise patient care and improve health outcomes; however, guidelines alone are insufficient to ensure changes in practice. This overview of reviews aims to identify, map and report on the effectiveness of knowledge translation (KT) strategies to implement guideline recommendations.

**Methods:**

We will conduct this study in accordance with the Cochrane Handbook guidance on overviews of reviews. We will search MEDLINE, CRD, SCOPUS, Cochrane Library, Web of Science, Health Systems Evidence, Epistemonikos, CINAHL and Embase. Eligible studies will include systematic reviews evaluating KT interventions for guideline implementation, regardless of the healthcare condition or system level. Teams of reviewers will independently screen and extract data on systematic review characteristics, interventions, outcomes (process‐, healthcare professional‐, patient‐ and economic‐related and other), contextual factors and review limitations. The same reviewers will assess risk of bias among included reviews using A MeaSurement Tool to Assess Systematic Reviews 2 (AMSTAR 2). We will synthesise the evidence by classifying intervention effectiveness into the following categories: (1) desirable effect, (2) little or no effect, (3) undesirable effect and (4) uncertain effect, along with the certainty of evidence, if reported. We will present our findings through a narrative synthesis, tabular formats, and an evidence gap map to visualise the evidence landscape.

**Discussion:**

This overview of reviews will provide a summary of KT strategies and their effectiveness for improving guideline implementation. These findings will offer actionable guidance for key interest‐holders: investigators can utilise the evidence gap map to identify research priorities; guideline developers can identify effective strategies for increasing uptake of recommendations; and funders can optimise resource allocation by directing support towards evidence‐based dissemination strategies.

## Summary

- **What Is Already Known on This Topic?**
  ◦ Health guidelines are necessary to optimise patient care; however, their publication alone does not guarantee change in practice.
  ◦ Knowledge translation (KT) strategies aim to facilitate the implementation of guideline recommendations.
  ◦ The most recent overview of reviews on this topic searches literature only up to 2019 and utilised methodological approaches that deviated from current best practices, necessitating an update.

- **What This Study Adds?**
  ◦ The overview of reviews outlined in this protocol will identify, map, and evaluate the effectiveness of KT strategies for implementing guideline recommendations across individual, organisational and system levels.
  ◦ This study will generate an evidence gap map and an intervention‐outcome matrix to clearly visualise the landscape of evidence, distinguishing between effective strategies and areas where evidence is uncertain.
  ◦ These findings will offer actionable guidance to assist interest‐holders, including guideline developers and funders, in selecting evidence‐informed strategies to ensure the uptake of recommendations.

## 1 Background

Health care guidelines are defined as ‘systematically developed statements to assist practitioner and patient decisions about appropriate health care for specific clinical circumstances’ [1]. The Canadian Institutes of Health Research knowledge to action framework acknowledges guidelines as essential tools for optimising patient care [2]. They serve as an important product to bridge research and practice [2] and support clinicians, patients and their caregivers and decision makers in navigating the benefits and harms of available interventions [1].

Although guidelines are necessary to inform evidence‐based care, their presence alone does not guarantee changes in clinical practice [3]. Further, it takes an average of 17 years for evidence to translate into practice [4]. Knowledge translation (KT) involves dynamic and iterative processes of synthesising, disseminating, exchanging and ethically applying knowledge to enhance health outcomes, service delivery and health system performance [5]. KT strategies can target various health system levels (i.e., individual‐level interactions [micro], institutional policies [meso] and societal factors [macro]) [6]. Integrating guidelines into national policies or reimbursement schemes (macro level systems) can promote sustained adoption and, for strong recommendations, quality assurance [7, 8, 9, 10].

The effectiveness of guideline implementation is influenced by contextual moderators, which are specific features of a healthcare environment, involved professionals and relevant patient populations. The impact of these contextual factors can be illustrated by the interactions across different health system levels. For example, a clinical practice guideline from a national society (macro level) promoting rapid thrombolysis for acute ischaemic stroke may be difficult to implement if a hospital (meso level) lacks the resources for 24‐h diagnostic imaging or an established ‘code stroke’ protocol [11]. Successful implementation can also be hindered at the micro level if frontline clinical teams are not sufficiently trained on the protocol [11]. A thorough understanding of this interplay is important for selecting and tailoring KT strategies effectively.

In 2022, an overview of reviews identified 36 systematic reviews assessing 30 KT strategies across conditions and health system levels, but these have several limitations [12]. First, the search was last conducted in 2019 [12], and several key developments in the field of KT have emerged over the past 6 years, particularly—driven by the Coronavirus disease 2019 (COVID‐19) pandemic—the accelerated adoption of digital health technologies such as telemedicine and mobile health applications [13]. Second, the methodology used in the previous overview [12] diverged from the guidance outlined in Chapter V of the Cochrane Handbook for Overviews of Reviews [14] in several important ways, including: (1) re‐extracting data from primary studies; (2) categorising interventions as effective, mixed, or ineffective based on a vote counting approach (i.e., tallying statistically significant findings), which disregards effect sizes, study weights and confidence intervals and (3) failing to report whether GRADE certainty of evidence assessments were conducted by the individual reviews.

Our overview of reviews aims to address these limitations and comprehensively identify, map, and report on the effectiveness of KT strategies used in guideline implementation. Our findings will support guideline developers, KT specialists and funders in selecting evidence‐informed strategies to improve guideline dissemination and implementation.

## 2 Research Question

What KT strategies have been used to facilitate implementation of health‐related guidelines across healthcare settings, and what is known about their effectiveness?

## 3 Methods

We developed our protocol following guidance from the Cochrane Handbook chapter on Overviews of Reviews [14]. We adhered to the preferred reporting items for systematic reviews and meta‐analyses protocols (PRISMA‐P) checklist [15] (Supporting Information: Table S1), and when reporting our findings, we will adhere to the Preferred Reporting Items for Overviews of Reviews (PRIOR) [16] checklist.

### 3.1 Eligibility Criteria

We will apply the following eligibility criteria.

#### 3.1.1 Population

We will place no restrictions on the population. Eligible reviews may include, but are not limited to, those targeting healthcare professionals, healthcare teams, organisations, policymakers or patients.

#### 3.1.2 Intervention

We will include reviews that evaluate the effectiveness of any KT strategy, whether single or multifaceted, intended to implement guideline recommendations for any health‐related condition or disease.

#### 3.1.3 Comparator

We will include reviews that include studies where the comparison is an alternative KT strategy or no strategy.

#### 3.1.4 Outcome

All outcomes reported by the review pertaining to effectiveness will be included.

***Setting:*** We will place no restrictions on the setting. Reviews will be eligible regardless of the healthcare system level, which may include the macro (e.g., international, national), meso (e.g., regional, healthcare organisation) and micro (e.g., healthcare professionals or teams) levels.

***Study Design:*** We will include systematic reviews, including mixed‐methods reviews, published in any language. The included systematic reviews may contain primary studies of any design. To be eligible for inclusion, a report must meet our operational definition of a systematic review, which includes the following three components:

1. Presents a clearly‐defined research question.
2. Documents systematic processes for study identification and selection.
3. Reports an assessment of the methodological quality of included studies.

If a systematic review has been updated, we will only include the most recent version.

#### 3.1.5 Exclusion

We will exclude primary studies, conference abstracts, meeting proceedings, editorials and commentaries.

### 3.2 Search Methods for Identification of Reviews

An experienced medical librarian (R.J.C.) will update the search strategy used in the previous overview of reviews [12], which has been peer‐reviewed, applying it across the same nine databases: MEDLINE (Ovid), CRD, SCOPUS (Elsevier), Cochrane Library (Wiley), Web of Science (Clarivate), Health Systems Evidence, Epistemonikos, CINAHL (EBSCO) and Embase (Ovid). We will not apply language restrictions to the search. Each database‐specific search strategy is provided in Supporting Information: Table S2. As this is an update to the overview by Pereira et al. [12], which searched until 2019, our search will cover the period from 2019 to the present.

### 3.3 Study Selection

We will screen all citations identified through the searches using the Covidence software (https://www.covidence.org/). Before initiating the screening process, teams of two reviewers will undergo training and calibration exercises using standardised and pre‐tested forms. They will then screen, independently, the titles and abstracts of all unique citations and the retrieved full texts of all articles judged as potentially eligible by at least one reviewer. The reviewers will resolve disagreements through discussion or with a third, senior reviewer when required. Papers in languages other than English will be assessed for eligibility by team members with language proficiency and the translation software DeepL (https://www.deepl.com/en/translator) where needed.

### 3.4 Data Abstraction

Teams of two reviewers will independently abstract data into a standardised data extraction form using Excel. Supporting Information: Table S3 provides a full list of data extraction variables.

From each eligible systematic review, we will extract data on:

1. Review characteristics: including title, first author, publication year, date of the last update, number of included studies, risk of bias tool and if review used GRADE;
2. Primary study characteristics included in the reviews such as study designs, populations studied and relevant contextual factors. Contextual factors include the country and income level, the specific healthcare setting (e.g., primary care, hospital), the target audience (e.g., physicians, patients), the clinical area and any other relevant factors reported by the review, such as cross‐health‐system level influences.
3. KT Interventions: including types of KT strategies (single or multifaceted), comparators (when applicable) (e.g., no intervention, usual care, alternative KT strategies), duration of KT strategy delivery and level of implementation (i.e., micro, meso, macro). We will classify interventions according to the system developed by Pereira et al. as described in Supporting Information: Table S4 [12]. This classification system was primarily based on the Cochrane Effective Practice and Organisation of Care (EPOC) taxonomy [12]. When certain KT interventions did not align with existing EPOC categories, the authors used additional categories from Grimshaw et al. [12, 17]. If KT strategies still did not fit within either set, they developed new categories iteratively to ensure comprehensive coverage [12].
4. Outcomes: We will classify the outcomes extracted into one of five categories: (1) process‐related outcomes (e.g., extent of guideline adherence in practice); (2) healthcare professional outcomes (e.g., knowledge, attitudes, behaviours); (3) patient‐outcomes (e.g., effects on health status, quality of life, satisfaction); (4) economic outcomes (e.g., cost effectiveness, financial implications) and (5) other outcomes (i.e., any outcome reported by an included systematic review that did not fit into the above categories).
5. Key findings: both narrative summaries and results from any meta‐analyses performed within included reviews, and GRADE certainty ratings (if applicable); and
6. Review limitations, conflicts of interest disclosures and funding sources when available.

### 3.5 Methodological Assessment of Included Reviews

Two reviewers will independently assess the methodological quality of included systematic reviews using the A MeaSurement Tool to Assess Systematic Reviews 2 (AMSTAR 2) tool [18]. To ensure consistency, we will conduct calibration exercises on a sample of included reviews prior to beginning formal assessment. Reviewers will resolve disagreements that remain after discussion with the help of a senior reviewer. The AMSTAR 2 tool evaluates systematic reviews of randomised and nonrandomised studies and includes 16 items across several domains as described in Supporting Information: Table S3. AMSTAR 2 does not generate a numeric score but rather categorises reviews based on critical methodological weaknesses and rates the review as being of high confidence if there are no or one noncritical weakness, moderate confidence if there is more than one noncritical weakness, low confidence if there is one critical flaw (with or without noncritical weaknesses) and critically low confidence if there is more than one critical flaw (with or without noncritical weaknesses).

For mixed‐methods systematic reviews (MMSRs), we will apply an appraisal approach by Jimenez et al., 2018 [19]. This includes evaluating: (1) the rigour of the quantitative and qualitative components, and (2) the quality of integration across stages of the review (e.g., theory of change development, synthesis and interpretation).

### 3.6 Data Synthesis

We will use the kappa statistic (*κ*) to measure inter‐rater reliability of reviewers for both the title and abstract and full‐text screening [20].

We will present the synthesis by first describing the characteristics of the included systematic reviews, their primary studies and their methodological quality based on our AMSTAR 2 assessments. We will generate two distinct summary outputs presented in tabular and narrative formats.

First, we will construct an evidence gap map to visualise the evidence landscape, including all systematic reviews regardless of their AMSTAR II rating. The map's rows will represent KT strategies and columns will represent outcome categories, with each cell indicating the volume of evidence (number of systematic reviews) and a summary of the reviews’ trustworthiness (via AMSTAR 2). Second, we will provide a detailed intervention‐outcome matrix presenting the most reliable evidence. This matrix will focus exclusively on findings from reviews with moderate or high AMSTAR 2 ratings. In a supplementary file, we will provide a descriptive summary of findings from reviews rated as low or critically low.

To populate the intervention‐outcome matrix, we will assess effectiveness for each KT strategy‐outcome pair by classifying the findings into one of four categories: (1) desirable effect, (2) little or no effect, (3) undesirable effect and (4) uncertain effect. This classification will be presented alongside the GRADE certainty of evidence when reported. We will explicitly note when a systematic review does not report the certainty of evidence.

To complement the matrix, our primary narrative synthesis, presented by KT strategy, will also focus on the findings from reviews with moderate or high trustworthiness. For each strategy, we will describe the evidence landscape by summarising the contextual characteristics of the supporting reviews. When reported by a systematic review, we will link the findings directly to the context in which they were observed and report on any explicit findings related to cross‐level influences across the healthcare system levels (e.g., a macro‐level policy impacting effectiveness of a meso‐level program). A dedicated section will be used to synthesise multifaceted interventions, where we will report on the effectiveness of the intervention bundle as a whole and descriptively group strategies that share common components.

As our overview addresses a broad topic rather than a narrow PICO question, we will not re‐analyze primary study data.

In accordance with Cochrane guidance for overviews of systematic reviews, we will retain all eligible systematic reviews, including those with overlapping primary studies [14]. This ensures a comprehensive mapping of the existing evidence base on KT interventions. To assess and transparently report overlap of primary studies between reviews, we will construct a citation matrix, mapping primary studies across included reviews and calculate the corrected covered area (CCA) to quantify the extent of overlap [21, 22]. We will also conduct this assessment for subgroups of reviews addressing the same KT strategy category. We will interpret the magnitude of overlap using established thresholds (slight: 0%–5%, moderate: 6%–10%, high: 11%–15%, very high: > 15%) [23].

## 4 Discussion

This protocol outlines a systematic approach to identifying, mapping and describing systematic review evidence on KT strategies for guideline implementation. Our findings will provide a rigorous and transparent overview of the effectiveness of KT interventions for guideline implementation across macro, meso and micro healthcare system levels.

### 4.1 Strengths and Limitations

Our overview has several methodological strengths. First, our adherence to Cochrane guidance on overviews of reviews [14], including appraising the quality of included reviews and reporting GRADE assessments where available. Second, we will conduct comprehensive searches with no language restrictions and include reviews with a systematic search strategy and a clear description of their risk of bias assessment for primary studies. Third, we will classify KT interventions according to current best practices [12, 23].

Our overview has two main limitations. First, the findings are constrained by the methodological quality of the included systematic reviews. Second, our eligibility criteria exclude relevant primary studies on KT strategies that have not yet been included and synthesised in a systematic review.

### 4.2 Implications for Relevant Interest‐Holders

Our findings are intended to provide actionable insights for key interest‐holders.

#### 4.2.1 For Researchers

The evidence gap map will allow researchers to identify specific gaps in the literature to focus future studies. For guideline developers, the intervention‐outcome matrix will assist in selecting effective strategies for guideline implementation. Further, our findings will support future development of a decision support tool to guide optimal selection and tailoring of these strategies.

#### 4.2.2 For Decision‐Makers

Funders can utilise findings on effectiveness to determine which strategies to support and which to discontinue, while using the gap map to prioritise funding for under‐researched areas. Policymakers can use findings regarding meso‐ and macro‐level interventions to inform resource allocation and health policy decisions.

## 5 Conclusion

This overview will comprehensively map KT strategies described in existing systematic reviews and provide critical insights for interest holders. By synthesising and evaluating the current evidence, our findings will help clarify which KT strategies are effective, which are not, and where the evidence remains uncertain. This will support more informed decision‐making and strategic planning for the implementation and uptake of guidelines across diverse healthcare settings.

## Author Contributions

**Kian Torabiardakani:** conceptualisation, methodology, writing – original draft; writing – review and editing. **Shraddha Sawant:** writing – review and editing. **Gonzalo Bravo‐Soto:** writing – review and editing. **Daniela Montalva‐Romero:** writing – review and editing. **Rachel J. Couban:** writing – review and editing. **Abigail E. Chu:** writing – review and editing. **Norman Buckley:** writing – review and editing. **Gordon Guyatt:** writing – review and editing. **David Juurlink:** writing – review and editing. **Jason W. Busse:** conceptualisation, writing – review and editing. **Andrea J. Darzi:** conceptualisation, methodology, writing – original draft, writing – review and editing.

## Ethics Statement

The authors have nothing to report.

## Conflicts of Interest

The authors declare no conflicts of interest.

## Guarantor

Kian Torabiardakani and Andrea J. Darzi are the guarantors for this paper.

## Supporting information

Final_file.

## Data Availability

All materials are presented within the protocol.
