# Supplementary material for: Effectiveness of Knowledge Translation Strategies for Guideline Implementation: Protocol for an Update of an Overview of Systematic Reviews
Source: Clin Public Health Guidel. 2026 Jan 28;3(2):e70058. doi: 10.1002/gin2.70058 (PMC13563359; doi:10.1002/gin2.70058)
Supplement: Supplementary file 1 [file GIN2-3-e70058-s001.docx]

**Supplementary Materials**

***e-Table 1:*** *Preferred Reporting Items for Systematic Reviews and Meta-Analyses Protocols (PRISMA-P) checklist*

***e-Table 2:*** *Search strategy*

***e-Table 3:*** *Data extraction variables & AMSTAR 2 items*

***e-Table 4:*** *Classification of implementation strategies, adapted from the Pereira et al., overview of reviews*

***e-Table 1:*** *Preferred Reporting Items for Systematic Reviews and Meta-Analyses Protocols (PRISMA-P) checklist*

**PRISMA-P 2015 Checklist**

# **Reference: Moher D, Shamseer L, Clarke M, Ghersi D, Liberati A, Petticrew M, Shekelle P, Stewart LA, Prisma-P Group. Preferred reporting items for systematic review and meta-analysis protocols (PRISMA-P) 2015 statement. Systematic reviews. 2015 Dec;4:1-9.**

| **Section/topic** | **#** | **Checklist item** | **Information reported** | | **Line number(s)** |
| --- | --- | --- | --- | --- | --- |
|  |  |  | **Yes** | **No** |  |
| **ADMINISTRATIVE INFORMATION** | | | | | |
| **Title** | | | | | |
| Identification | 1a | Identify the report as a protocol of a systematic review | ☐ | ☐ | Lines 1 to 2 |
| Update | 1b | If the protocol is for an update of a previous systematic review, identify as such | ☐ | ☐ | Lines 1 to 2 |
| **Registration** | 2 | If registered, provide the name of the registry (e.g., PROSPERO) and registration number in the Abstract | ☐ | ☐ | Not applicable as submitted to a journal. |
| **Authors** 37 to 45) | | | | | |
| Contact | 3a | Provide name, institutional affiliation, and e-mail address of all protocol authors; provide physical mailing address of corresponding author | ☐ | ☐ | Lines 4 to 29 |
| Contributions | 3b | Describe contributions of protocol authors and identify the guarantor of the review | ☐ | ☐ | Lines 37 to 45 |
| **Amendments** | 4 | If the protocol represents an amendment of a previously completed or published protocol, identify as such and list changes; otherwise, state plan for documenting important protocol amendments | ☐ | ☐ | Not applicable |
| **Support** | | | | | |
| Sources | 5a | Indicate sources of financial or other support for the review | ☐ | ☐ | Line 32 |
| Sponsor | 5b | Provide name for the review funder and/or sponsor | ☐ | ☐ | Not applicable |
| Role of sponsor/funder | 5c | Describe roles of funder(s), sponsor(s), and/or institution(s), if any, in developing the protocol | ☐ | ☐ | Lines 32 to 35 |
| **INTRODUCTION** | | | | | |
| **Rationale** | 6 | Describe the rationale for the review in the context of what is already known | ☐ | ☐ | Lines 87 to 103 |
| **Objectives** | 7 | Provide an explicit statement of the question(s) the review will address with reference to participants, interventions, comparators, and outcomes (PICO) | ☐ | ☐ | Line 99 to 101 |
| **METHODS** | | | | | |
| **Eligibility criteria** | 8 | Specify the study characteristics (e.g., PICO, study design, setting, time frame) and report characteristics (e.g., years considered, language, publication status) to be used as criteria for eligibility for the review | ☐ | ☐ | Lines 105 to 141 |
| **Information sources** | 9 | Describe all intended information sources (e.g., electronic databases, contact with study authors, trial registers, or other grey literature sources) with planned dates of coverage | ☐ | ☐ | Lines 143 to 148. |
| **Search strategy** | 10 | Present draft of search strategy to be used for at least one electronic database, including planned limits, such that it could be repeated | ☐ | ☐ | e-Table 3 |
| ***STUDY RECORDS*** | | | | | |
| Data management | 11a | Describe the mechanism(s) that will be used to manage records and data throughout the review | ☐ | ☐ | Lines 150 to 156 |
| Selection process | 11b | State the process that will be used for selecting studies (e.g., two independent reviewers) through each phase of the review (i.e., screening, eligibility, and inclusion in meta-analysis) | ☐ | ☐ | Lines 150 to 156 |
| Data collection process | 11c | Describe planned method of extracting data from reports (e.g., piloting forms, done independently, in duplicate), any processes for obtaining and confirming data from investigators | ☐ | ☐ | Lines 150 to 156 |
| **Data items** | 12 | List and define all variables for which data will be sought (e.g., PICO items, funding sources), any pre-planned data assumptions and simplifications | ☐ | ☐ | Lines 158 to 175 |
| **Outcomes and prioritization** | 13 | List and define all outcomes for which data will be sought, including prioritization of main and additional outcomes, with rationale | ☐ | ☐ | Lines 171-172 |
| **Risk of bias in individual studies** | 14 | Describe anticipated methods for assessing risk of bias of individual studies, including whether this will be done at the outcome or study level, or both; state how this information will be used in data synthesis | ☐ | ☐ | Lines 177 to 187 |
| ***DATA*** | | | | | |
| **Synthesis** | 15a | Describe criteria under which study data will be quantitatively synthesized | ☐ | ☐ | Lines 192 to 212 |
|  | 15b | If data are appropriate for quantitative synthesis, describe planned summary measures, methods of handling data, and methods of combining data from studies, including any planned exploration of consistency (e.g., *I* ^2^, Kendall’s tau) | ☐ | ☐ | Lines 192 to 212 |
|  | 15c | Describe any proposed additional analyses (e.g., sensitivity or subgroup analyses, meta-regression) | ☐ | ☐ | Not applicable, as overview of review |
|  | 15d | If quantitative synthesis is not appropriate, describe the type of summary planned | ☐ | ☐ | Lines 192 to 212 |
| **Meta-bias(es)** | 16 | Specify any planned assessment of meta-bias(es) (e.g., publication bias across studies, selective reporting within studies) | ☐ | ☐ | Lines 210 to 212 |
| **Confidence in cumulative evidence** | 17 | Describe how the strength of the body of evidence will be assessed (e.g., GRADE) | ☐ | ☐ | Line 165 |

***e-Table 2:*** *Search strategy*

The search strategy from Pereira et al.'s overview of reviews (11) has been updated for this study, extending the search beyond 2019.

**Table 1.** Databases searched and number of studies retrieved (2019 till November 11^th^, 2024) **^*^**

| **Databases** | **Total** |
| --- | --- |
| MEDLINE | 958 |
| CRD | 0 |
| SCOPUS | 1,182 |
| Cochrane | 383 |
| Web of Science | 890 |
| Rx for Change**^**^** | NA |
| Health Systems Evidence | 108 |
| Epistemonikos | 79 |
| CINAHL | 742 |
| Embase | 9,946 |
| *Subtotal* | 14,288 |
| *Duplicates* | 2,653 |
| *Total* | 11,635 |

**^*^**The search strategy was initially conducted on February 27^th^, 2024, and updated between October 31^st^ and November 11^th^, 2024. Next page you will find the specific date each database was searched during the update.

**^**^**The Canadian Agency for Drugs and Technologies in Health (CADTH) was contacted regarding the Rx for Change database, and it was confirmed that the database is no longer active or available for search.

MEDLINE (OVID)

Database: OVID Medline Epub Ahead of Print, In-Process & Other Non-Indexed Citations, Ovid MEDLINE(R) Daily and Ovid MEDLINE(R) 1946 to Present (October 31^st^, 2024)

Search Strategy:

**--------------------------------------------------------------------------------**

1	Practice Guidelines as Topic/ (130318)

2	practice guideline.pt. (32509)

3	Clinical Protocols/ (30268)

4	Critical Pathways/ (8150)

5	Health Planning Guidelines/ (4165)

6	or/1-5 (200896)

7	Guideline Adherence/ (35905)

8	Health Plan Implementation/ (6650)

9	(implement* or aware* or uptake or up-take or take up or take-up or adhere or adhered or adherence or concordance or accordance or adopt* or comply or complies or compliance or disseminat* or spread or spreading or barrier* or facilitat*).mp. (3795275)

10	or/7-9 (3795275)

11	6 and 10 (54578)

12	meta-analysis.pt. (210481)

13	Meta-Analysis as Topic/ (24769)

14	meta-analysis.ti,ab. (275863)

15	(systematic and review).ti,ab. (384067)

16	or/12-15 (537637)

17	(case and report).ti. (397637)

18	editorial.pt. (711149)

19	comment.pt. (1044151)

20	newspaper article.pt. (18297)

21	letter.pt. (1277745)

22	or/17-21 (2693044)

23	16 not 22 (518380)

24	11 and 23 (2841)

25	limit 24 to yr="2019 -Current" (958)

26	limit 25 to ed=20240227-20241031 (147)

27	limit 25 to dt=20240227-20241031 (105)

28	26 or 27 (155)

CRD

**--------------------------------------------------------------------------------**

|  | Line | Search | Hits |
| --- | --- | --- | --- |
|  | 1 | MeSH DESCRIPTOR Practice Guidelines as Topic EXPLODE ALL TREES | 791 |
|  | 2 | MeSH DESCRIPTOR Clinical Protocols EXPLODE ALL TREES | 1296 |
|  | 3 | MeSH DESCRIPTOR Health Planning Guidelines EXPLODE ALL TREES | 22 |
|  | 4 | MeSH DESCRIPTOR Critical Pathways EXPLODE ALL TREES | 150 |
|  | 5 | #1 OR #2 OR #3 OR #4 | 2224 |
|  | 6 | MeSH DESCRIPTOR Guideline Adherence EXPLODE ALL TREES | 154 |
|  | 7 | MeSH DESCRIPTOR Health Plan Implementation EXPLODE ALL TREES | 18 |
|  | 8 | (implement* or aware* or uptake or up-take or take up or take-up or adhere or adhered or adherence or concordance or accordance or adopt* or comply or complies or compliance or disseminat* or spread or spreading or barrier* or facilitat*) | 13931 |
|  | 9 | #6 OR #7 OR #8 | 13931 |
|  | 10 | #5 AND #9 | 563 |
|  | 11 | (#10) FROM 2019 TO 2024 | 0 |

Scopus (Elsevier)

**--------------------------------------------------------------------------------**

( TITLE-ABS-KEY ( "practice guideline" OR "practice guidelines" OR "clinical protocol" OR "clinical protocol" OR "health planning guideline" OR "health planning guidelines" OR "critical pathway" OR "critical pathways" ) ) AND ( TITLE-ABS-KEY ( implementation OR adherence ) ) AND ( TITLE ( meta-analysis OR "systematic review" ) ) AND PUBYEAR = 2024

Cochrane (Wiley)

Search Name:

Date Run:	01/11/2024 19:04:58

**--------------------------------------------------------------------------------**

ID	Search	Hits

#1	MeSH descriptor: [Practice Guidelines as Topic] explode all trees	3200

#2	"practice guidelines" or "practice guideline" or "clinical protocol" or "clinical protocols" or "critical pathways" or "critical pathway"	31827

#3	#1 or #2	31827

#4	"Guideline Adherence" OR "Health Plan Implementation" OR implement* or aware* OR uptake OR up-take OR “take up” OR take-up OR adhere OR adhered OR adherence OR concordance OR accordance OR adopt* OR comply or complies or compliance or disseminat* OR spread OR spreading OR barrier or barriers OR facilitat*	298932

#5	#3 and #4 with Cochrane Library publication date Between Jan 2019 and Nov 2024, in Cochrane Reviews	383

#6	#5 with Cochrane Library publication date Between Feb 2024 and Nov 2024	45

Web of Science (Clarivate)

**--------------------------------------------------------------------------------**

1 "practice guidelines" or "clinical protocols" or "critical pathways" or "health planning guidelines" (Topic) 52,794

2 TS=("guideline adherence" or "Health plan implementation" or implement* or aware* or uptake or up-take or "take up" or take-up or adhere or adhered or adherence or concordance or accordance or adopt* or comply or complies or compliance or disseminat* or spread or spreading or barrier* or facilitat*) 8,158,987

3 TS=(meta-analysis or "meta analysis" or "systematic review") 567,463

4 #1 AND #2 AND #3 1,460

5 #4 date limit 2024-02-27 to 2024-11-01 131

6 #4 date limit 2019-01-01 to 2024-11-01 890

Health Systems Evidence

**--------------------------------------------------------------------------------**

Search: guideline implementation

Epistemonikos

**--------------------------------------------------------------------------------**

title:((Practice Guideline*) OR (Clinical Protocol*) OR (Critical Pathway*) OR

(Health Planning Guideline*)) AND title:((Guideline Adherence) OR (Health Plan

Implementation) OR implement* OR aware* OR uptake OR up-take OR "take up"

OR take-up OR adhere OR adhered OR adherence OR concordance OR accordance

OR adopt* OR comply OR complies OR compliance OR disseminat* OR spread OR

spreading OR barrier* OR facilitat*) AND (title:("meta-analysis" OR "meta

analysis" OR "systematic review") OR abstract:("meta-analysis" OR "meta

analysis" OR "systematic review")) year=2024

CINAHL (EBSCO)

**--------------------------------------------------------------------------------**

Wed, November 6, 2024 2:31:45 PM

| **#** | **Query** | **Limiters/Expanders** | **Last Run Via** | **Results** |
| --- | --- | --- | --- | --- |
| S16 | S14 | Limiters - Publication Date: 20190101-20241231 Search modes - Proximity | Interface - EBSCOhost Research Databases Search Screen - Advanced Search Database - CINAHL | 742 |
| S15 | S14 | Limiters - Publication Date: 20240201-20241131 Search modes - Proximity | Interface - EBSCOhost Research Databases Search Screen - Advanced Search Database - CINAHL | 86 |
| S14 | S12 AND S13 | Search modes - Proximity | Interface - EBSCOhost Research Databases Search Screen - Advanced Search Database - CINAHL | 1,762 |
| S13 | TX meta-analysis or "meta analysis" or "systematic review" | Search modes - Proximity | Interface - EBSCOhost Research Databases Search Screen - Advanced Search Database - CINAHL | 256,001 |
| S12 | S6 AND S11 | Search modes - Proximity | Interface - EBSCOhost Research Databases Search Screen - Advanced Search Database - CINAHL | 27,514 |
| S11 | S7 OR S8 OR S9 OR S10 | Search modes - Proximity | Interface - EBSCOhost Research Databases Search Screen - Advanced Search Database - CINAHL | 890,156 |
| S10 | AB implement* or aware* or uptake or up-take or "take up" or take-up or adhere or adhered or adherence or concordance or accordance or adopt* or comply or complies or compliance or disseminat* or spread or spreading or barrier* or facilitat* | Search modes - Proximity | Interface - EBSCOhost Research Databases Search Screen - Advanced Search Database - CINAHL | 867,815 |
| S9 | TI implement* or aware* or uptake or up-take or "take up" or take-up or adhere or adhered or adherence or concordance or accordance or adopt* or comply or complies or compliance or disseminat* or spread or spreading or barrier* or facilitat* | Search modes - Proximity | Interface - EBSCOhost Research Databases Search Screen - Advanced Search Database - CINAHL | 749,975 |
| S8 | "health plan implementation" | Search modes - Proximity | Interface - EBSCOhost Research Databases Search Screen - Advanced Search Database - CINAHL | 5 |
| S7 | (MH "Guideline Adherence") | Search modes - Proximity | Interface - EBSCOhost Research Databases Search Screen - Advanced Search Database - CINAHL | 18,164 |
| S6 | S1 OR S2 OR S3 OR S4 OR S5 | Search modes - Proximity | Interface - EBSCOhost Research Databases Search Screen - Advanced Search Database - CINAHL | 103,162 |
| S5 | AB practice guidelines | Search modes - Proximity | Interface - EBSCOhost Research Databases Search Screen - Advanced Search Database - CINAHL | 12,606 |
| S4 | "health planning guidelines" | Search modes - Proximity | Interface - EBSCOhost Research Databases Search Screen - Advanced Search Database - CINAHL | 0 |
| S3 | (MH "Critical Path") | Search modes - Proximity | Interface - EBSCOhost Research Databases Search Screen - Advanced Search Database - CINAHL | 6,218 |
| S2 | ""clinical protocols"" | Search modes - Proximity | Interface - EBSCOhost Research Databases Search Screen - Advanced Search Database - CINAHL | 1,511 |
| S1 | (MH "Practice Guidelines") | Search modes - Proximity | Interface - EBSCOhost Research Databases Search Screen - Advanced Search Database - CINAHL | 88,306 |

Embase (OVID)

Database: Embase <1974 to 2024 November 11>

Search Strategy:

**--------------------------------------------------------------------------------**

1	protocol compliance/ (23136)

2	(implement* or aware* or uptake or up-take or take up or take-up or adhere or adhered or adherence or concordance or accordance or adopt* or comply or complies or compliance or disseminat* or spread or spreading or barrier* or facilitat*).mp. (5016192)

3	1 or 2 (5016192)

4	exp practice guideline/ (768984)

5	("practice guideline" or "practice guidelines" or "clinical protocol").mp. or "critical pathway".ti,ab. (729323)

6	4 or 5 (791176)

7	3 and 6 (220704)

8	"systematic review"/ (494968)

9	meta analysis/ (335888)

10	8 or 9 (634667)

11	7 and 10 (15520)

12	*"systematic review"/ (11799)

13	*meta analysis/ (14578)

14	12 or 13 (22018)

15	7 and 14 (58)

16	limit 15 to dc=20240227-20241106 (0)

17	limit 15 to dd=20240227-20241106 (0)

18	16 or 17 (0)

19	limit 11 to dc=20240227-20241106 (1632)

20	limit 11 to dd=20240227-20241106 (655)

21	19 or 20 (1632)

22	limit 11 to yr="2019 -Current" (9931)

23	22 not 21 (8314)

24	21 or 22 or 23 (9946)

***e-Table 3:*** *Data extraction variables & AMSTAR 2 items*

| **Section** | **Data Field** | **Description/Notes** |
| --- | --- | --- |
| **Basic Review Information & Primary Study Characteristics** | Review Title | Title of the systematic review |
|  | First Author | First author of the review |
|  | Publication Year | Year of publication |
|  | Study design | Type of the study (e.g., RCT) |
|  | Date of Last Update | Date when the review was last updated |
|  | Number of Included Studies | Total number of studies included in the review |
|  | Number of Primary Studies | Number of primary studies included |
|  | Context (Countries/Regions) | Countries/regions where studies were conducted |
|  | Population Details | Key population characteristics (e.g., age, disease type) |
| **Interventions and Comparators** | KT Strategies | State the name of the KT strategy |
|  | Type of KT Strategy | Specify whether single or multifaceted |
|  | Detailed Description of KT Strategy | Detailed description of the KT strategy, including time frame if applicable |
|  | Comparator(s) | Description of comparators (e.g., no intervention, other KT activities) |
| **Reported Outcomes** | Process-Related Outcomes | Outcomes related to processes (e.g., guideline adherence) |
|  | Health Professional-Related Outcomes | Outcomes related to health professionals (e.g., knowledge) |
|  | Patient-Related Outcomes | Outcomes related to patients (e.g., health outcomes) |
|  | Economic Outcomes | Any economic outcomes (e.g., cost-effectiveness) |
|  | Other Outcomes | Any other reported outcomes |
| **Key Findings** | Narrative Summary of Results | Summary of key results from the review |
|  | Meta-Analysis Conducted | Was a meta-analysis conducted? |
|  | Pooled Effect Size | Effect sizes from the meta-analysis |
|  | Subgroup Analyses | Details of any subgroup analyses conducted |
| **Risk of Bias and Methodological Quality** | Risk of Bias Assessment Reported | Did the review assess risk of bias? |
|  | Key Findings of Risk of Bias | Summary of the risk of bias findings |
| **Additional Information** | Conflicts of Interest (Y/N) | Were conflicts of interest reported? |
|  | Funding Source | Funding source for the review |
| **AMSTAR 2 Assessment** | 1. PICO Components | Did the research questions and inclusion criteria include Population, Intervention, Comparator, and Outcome (PICO)? |
|  | 2. Protocol Established Before Review | Was there a protocol established prior to the review, and were deviations from it justified? |
|  | 3. Explanation of Study Designs | Did the review authors explain the selection of study designs for inclusion? |
|  | 4. Comprehensive Literature Search | Did the review authors use a comprehensive search strategy (e.g., searched multiple databases, grey literature)? |
|  | 5. Study Selection in Duplicate | Were at least two reviewers involved in study selection to achieve consensus? |
|  | 6. Data Extraction in Duplicate | Was data extraction performed by at least two reviewers to ensure consistency? |
|  | 7. List of Excluded Studies and Justification | Did the review authors provide a list of excluded studies with justifications for their exclusion? |
|  | 8. Adequate Description of Included Studies | Were the included studies described in sufficient detail (e.g., population, interventions, outcomes)? |
|  | 9. Risk of Bias Assessment | Did the review authors adequately assess the risk of bias in individual studies (e.g., allocation, blinding)? |
|  | 10. Sources of Funding for Included Studies | Did the review authors report the sources of funding for the included studies? |
|  | 11. Appropriate Statistical Combination (Meta-analysis) | If meta-analysis was performed, did the review authors use appropriate methods for statistical combination? |
|  | 12. Impact of Risk of Bias on Meta-Analysis | Did the review authors assess the impact of risk of bias on the results of the meta-analysis? |
|  | 13. Risk of Bias in Interpretation of Results | Did the review authors consider the risk of bias when interpreting and discussing the review's results? |
|  | 14. Explanation for Heterogeneity | If heterogeneity was present, did the review authors provide a satisfactory explanation for it? |
|  | 15. Assessment of Publication Bias | If quantitative synthesis was performed, did the review authors investigate and discuss the potential for publication bias? |
|  | 16. Conflict of Interest Reported | Did the review authors report any potential conflicts of interest, including funding sources for conducting the review? |
|  | Final Assessment | High Confidence: No or one non-critical weakness. Moderate Confidence: More than one non-critical weakness. Low Confidence: One critical flaw, with or without non-critical weaknesses. Critically Low Confidence: More than one critical flaw, with or without non-critical weaknesses. |

***e-Table 4:*** *Classification of implementation strategies, adapted from the Pereira et al., (11) overview of reviews*

| **Strategy** | **Code** | **Definition** |
| --- | --- | --- |
| **Coordination of care and management of care** | | |
| Care pathways | CAP | Aim to link evidence to practice for specific health conditions and local arrangements for delivering care |
| Case management | CAM | Introduction, modification, or removal of strategies to improve coordination and continuity of delivery of services |
| Clinical multidisciplinary teams | CMT | Creation of a new team of health professionals of different disciplines or addition of new members to the team |
| Communication between providers | CBP | Systems or strategies for improving communication between healthcare providers, e.g., immunization coverage systems |
| Continuity of care | COC | Interventions to reduce fragmented care, ensuring continuity across facilities for the patient’s needs and circumstances |
| **Information and communication technology** | | |
| Information and communication technology | ICT | ICT used by healthcare organizations to manage and deliver healthcare |
| **Changes to the healthcare environment** | | |
| Structural intervention | SI | Changes to the setting/site of service delivery, including physical structures, facilities, equipment, and medical records systems |
| **Authority and accountability for health policies** | | |
| Community mobilization | COM | Processes that enable people to organize among themselves |
| **Interventions targeted at healthcare workers** | | |
| Academic detailing | AD | Personal visits by a trained person to health workers in their settings, aiming to change practice |
| Audit and feedback | AF | Summary of health workers’ performance given in a written, electronic, or verbal format, including clinical recommendations |
| Communities of practice | CP | Groups of people with a common interest who interact to deepen their expertise |
| Continuous quality improvement | CQI | An iterative process for care improvement involving healthcare teams, process analysis, structured methods, and data assessment |
| Educational games | EG | Use of games as an educational strategy to improve care standards |
| Educational materials | EMA | Distribution of educational materials (printed, audiovisual, electronic) supporting clinical care |
| Educational meetings | EME | Courses, workshops, conferences, or other educational meetings |
| Local consensus processes | LCP | Formal or informal local consensus processes, such as agreeing on a clinical protocol or adapting a guideline for a local health system |
| Local opinion leaders | LOL | Identification and use of identifiable local opinion leaders to promote good clinical practice |
| Monitoring the performance of healthcare | MP | Monitoring health services by individuals or organizations, e.g., comparing with an external standard |
| Patient-mediated intervention | PMI | Interventions aimed at changing healthcare professional performance through patient interactions or information provided to patients |
| Reminders | RE | Manual or computerized prompts for health workers to perform actions during patient consultations, e.g., decision support systems |
| Tailored interventions | TI | Practice-changing interventions selected based on barrier assessments, such as through interviews or surveys |
| **Interventions targeted at healthcare organizations** | | |
| Organizational culture | ORG | Strategies to change organizational culture |
| Financial interventions | FI | Targeted financial incentives for healthcare professionals and organizations |
| Educational intervention | EI | Education-focused intervention |
| **Other strategies (not defined by EPOC nor the Grimshaw et al., review)** | | |
| Patient incentives | PIC | Financial reward or benefit provided to patients to encourage specific actions |
| Patient-directed interventions | PI | Interventions aimed at qualifying patients for self-care and decision-making |
| Administrative restriction | AR | Administrative restrictions related to prescriptions |
| Marketing | MKT | Standard business approaches used to encourage usage of materials |
| Mass media | MM | Use of communication channels like TV, radio, and print, alone or with other interventions, targeting a large population |
| Practice support | PS | Professional support available to aid clinical practice or direct patient support |
